# Carpometacarpal Dislocation with Third Metacarpal Fracture

**DOI:** 10.5811/cpcem.2021.9.53202

**Published:** 2021-11-01

**Authors:** Colin Jorgensen, Steve C. Christos

**Affiliations:** AMITA Health Resurrection Medical Center, Department of Emergency Medicine, Chicago, Illinois

**Keywords:** carpometacarpal dislocation, metacarpal fracture

## Abstract

**Case Presentation:**

A 17-year-old male presented to the emergency department (ED) due to trauma to the right hand and wrist after punching a locker at school. He had significant soft tissue swelling. Radiographs demonstrated intra-articular metacarpal fractures with associated carpometacarpal dislocations. The dislocation was reduced bedside in the ED and ultimately underwent closed reduction surgical management with orthopedic surgery.

**Discussion:**

Metacarpal fractures result from high-force impact injuries and account for 30–40% of all hand injuries. The most common sites of second through fifth metacarpal fractures are at the neck and the shaft, with the majority involving the fifth metacarpal neck (commonly coined “boxer’s fractures”). Carpometacarpal (CMC) dislocations are a rare injury associated with high-force impact trauma to the wrist. These injuries account for as little as 1% of all acute hand and wrist injuries.^1^,^2^ Carpometacarpal dislocations are often difficult to diagnose on physical examination due to significant soft tissue swelling, and they can easily be missed on anterior-posterior views of the hand. Lateral and oblique plain radiograph views are essential in the diagnosis as they are more likely to show dislocations. Despite appropriate plain radiographic views, subtle CMC dislocations may be difficult to discern dependent on the level of dislocation or subluxation and overlapping of joints. These injuries are rare due to otherwise highly stable ligamentous and muscular attachments within the wrist. Because of these attachments, dislocations are often associated with concomitant metacarpal fractures.^3^

## CASE PRESENTATION

A 17-year-old male presented to the emergency department (ED) with a chief complaint of right-hand pain after punching a locker with a closed fist. His vital signs were normal. Physical examination was notable for significant soft tissue swelling over the dorsum of the right hand with tenderness over the third through fifth metacarpals. The hand was neurovascularly intact with intact two-point discrimination. There was significantly limited range of motion at the metacarpophalangeal joints and interphalangeal joints. There were no open wounds. Plain film radiographs demonstrated comminuted intra-articular fractures through the radial aspect of the base of the third metacarpal with dorsal carpometacarpal (CMC) dislocations of the third through fifth metacarpals (Image).

A hematoma block was performed by injection of 10 cubic centimeters of 1:1 solution of 1% lidocaine and 0.5% bupivacaine. The CMC dislocation was reduced by applying axial traction at the metacarpal phalangeal joints with pressure at the dorsum of the proximal CMC joints. Post-reduction radiographs demonstrated successful reduction to anatomic alignment. The hand was immobilized, and the patient was advised to follow up with orthopedic surgery. He ultimately underwent closed reduction with percutaneous placement of Kirschner wires and was immobilized for six weeks.

## DISCUSSION

Metacarpal fractures are common traumatic hand injuries presenting to the ED; the most common site of second through fifth metacarpal fractures occur at the neck and shaft. First metacarpal fractures most commonly occur at the base and include Bennett (simple intra-articular fractures) and Rolando fractures (comminuted/complete intra-articular fractures at base of the first metacarpal). We report a rare third metacarpal base fracture with associated CMC dislocations at the third through fifth metacarpals. These are uncommon injuries due to the intrinsically stable nature of the CMC joints, particularly due to ligamentous insertion and muscular attachments. Dorsal dislocation is more common due to the relatively stronger dorsal interosseous ligaments compared to their volar counterparts.^3^

Diagnosis of CMC dislocations may be difficult due to significant soft tissue swelling and edema, as well as poorly visualized injury on anterior-posterior or posterior-anterior radiographs. While the dislocations are more readily apparent on lateral or oblique views, they are still often difficult to identify if there is minimal dorsal or volar displacement or if positioning does not provide adequate visualization. Management includes prompt identification of injury, manual reduction with immobilization, and early orthopedic surgery follow-up. Manual reduction is essential to providing initial care. Patients should be immobilized with 70–90^o^ of flexion at the MCP joint, slight flexion at the interphalangeal joints, and mild wrist extension.

Although there is discrepancy of thought regarding the necessity for surgery,^1^ it is generally definitively repaired surgically. Closed reduction with percutaneous Kirschner wire placement is the approach of choice. Although a primary ulnar nerve injury may be present, delayed treatment may result in neurovascular injury particularly in the presence of significant soft tissue swelling and edema. Additionally, instability at the joint may result in early-onset arthritis and articular degeneration.^4^ Delayed closed reduction or inability to successfully reduce the dislocation may result in need for an open reduction approach.^5^

**CPC-EM Capsule**

What do we already know about this clinical entity?

*Carpometacarpal (CMC) dislocations are rare wrist injuries. Manual reduction and prompt orthopedic follow-up are essential for management of these injuries.*

What is the major impact of the image(s)?

*This image demonstrates the importance of lateral and oblique radiographic views identifying CMC dislocations.*

How might this improve emergency medicine practice?

*By promptly identifying a CMC dislocation, the emergency clinician is better able to provide initial management of this rare traumatic injury.*

## Figures and Tables

**Image:**
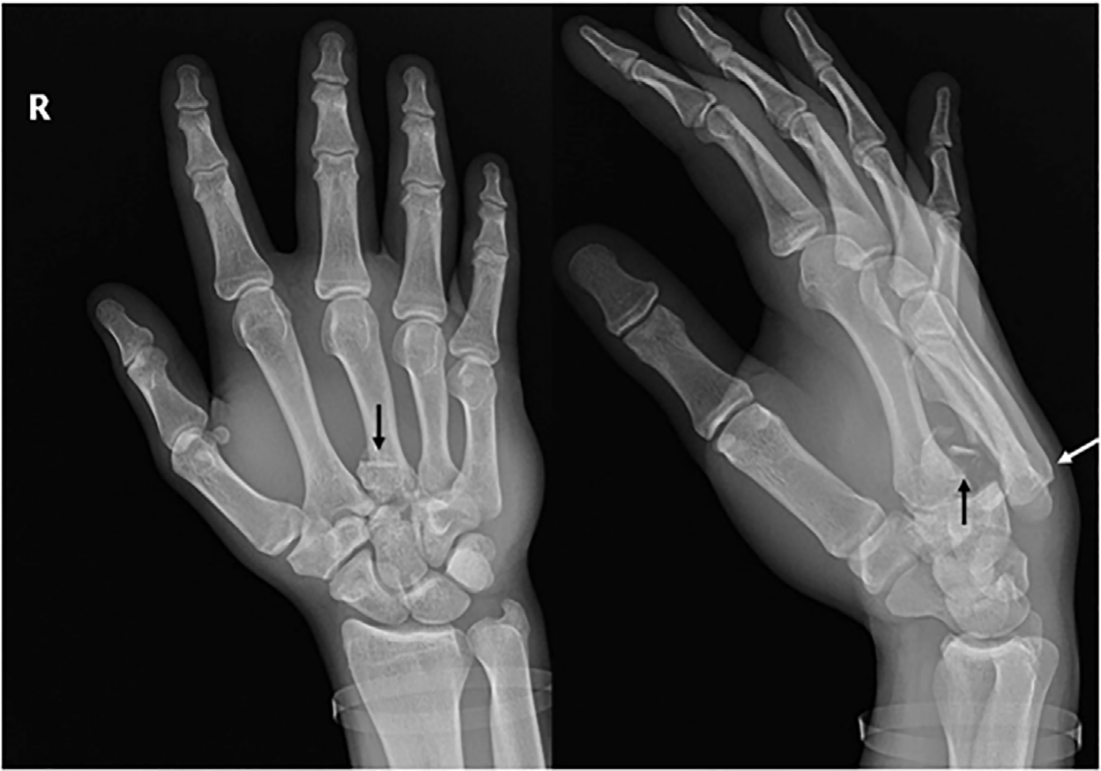
Anterior-posterior and oblique plain radiograph of the right hand demonstrates fractures through the radial aspect of the base of the third metacarpal (black arrows) with dorsal carpometacarpal dislocations of the third through fifth metacarpals (white arrow).
